# Bridging the Gap: Culturally Responsive Strategies for NIH Trial Recruitment

**DOI:** 10.1007/s40615-024-02166-y

**Published:** 2024-10-29

**Authors:** Lindsey Ross, Samuel Eberlein, Carine Khalil, So Yung Choi, Karma McKelvey, Brennan M. R. Spiegel

**Affiliations:** 1 Department of Neuroscience, Cedars-Sinai Medical Center, 129 S. San Vicente Blvd., A6600, Los Angeles, CA 90048, USA; 2 Division of Health Services Research Virtual Medicine Program, Cedars-Sinai Department of Medicine, Los Angeles, CA, USA; 3 Cedars-Sinai Biostatistics Shared Resource, Los Angeles, CA, USA; 4 Rocky Mountain College, Billings, MT, USA; 5 Division of Gastroenterology, Cedars-Sinai Department of Medicine, Los Angeles, CA, USA

**Keywords:** Health equity, Health disparities, Diversity, Clinical trials, Virtual reality, Digital health, Cohort builder

## Abstract

**Objective:**

To enhance recruitment and participation rates of non-Hispanic Black (NHB) and Hispanic adult patients in a NIH-funded clinical trial studying an emerging health technology.

**Data Sources and Study Setting:**

This study includes primary data collected in Los Angeles, California from November 2020 through November 2023.

**Study Design:**

To improve the representation of NHB and Hispanic patients in a NIH-funded (NCT04409353) trial on virtual reality for chronic lower back pain (cLBP), we conducted a multi-phase study utilizing a mixed-method approach. First, we conducted focus groups with NHB and Hispanic cohorts aged 18 and older; based on the feedback, we culturally adapted recruitment materials and study correspondences concordantly. Additionally, a cohort builder was used to filter the electronic medical record to isolate non-Hispanic Black (NHB) and Hispanic patients with cLBP for micro-targeted recruitment. These changes were collectively integrated when the parent NIH study had recruited 222 of its 385 final samples (57.7%), creating a pre-post comparison timepoint (May 17, 2022). Quantitative analysis was performed to assess the efficacy of the modified recruitment strategies by comparing the number of recruited and randomized NHB and Hispanic patients pre- and post-intervention.

**Data Collection/Extraction Methods:**

Semi-structured focus groups were conducted with NHB and Hispanic patients and community members (age 18 and older). The focus groups were conducted online and recorded with participant consent; transcripts of the recording underwent inductive thematic analysis. Emergent themes directed the modification of study materials, including revised language and imagery, targeted outreach, and incorporation of treating physicians, were implemented in the second half of the study. Quantitative analyses were conducted following parent study completion by comparing records added to the screening database following the implementation of new recruiting methods (5/17/2022) to those added before.

**Principal Findings:**

Thematic analysis of focus groups identified four key themes: mistrust, lack of interest, culture, and communication. Modifications to recruitment methodology resulted in statistically significant increases in the pre- to post-randomization success rate for the overall study population (*p* < 0.001), the NHB population (*p* = 0.011), and the Hispanic population (*p* < 0.015). When looking at each cohort at different points in the recruitment process before and after the intervention, in the Hispanic population, we saw significant increases in the number approached (*p* < 0.001) and number randomized (*p* < 0.001) and statistically insignificant increases in the NHB population approached (*p* = 0.067) and randomized (*p* = 0.295). Similarly, we saw that the changes in the recruitment letter led to a statistically significant increase in Hispanic recruitment (7.0 to 39.1%, *p* < 0.001) but not the NHB cohort (19.6 to 35.8%, *p* < 0.065).

**Conclusion:**

This study introduces several culturally sensitive considerations and possible approaches for the design of recruitment materials, addressing mistrust, lack of interest, culture, and communication for use in NHB and Hispanic populations. Similarly, the described microtargeting techniques leverage the technological advancements in cohort building to improve the reach and efficiency of the randomization rate of underrepresented groups thereby enhancing clinical trial diversity.

## Introduction

Diversity in research representation is crucial for enhancing healthcare practice. The lack of diversity in clinical trials worsens the generalizability of research findings and perpetuates health and healthcare disparities [1]. Despite the prioritization of diversity by organizations such as the Institute of Medicine (IOM) and National Institutes of Health (NIH), clinical trials in the USA continue to be predominantly White. Estimates suggest that 75 to 90% of trial participants identify as White [2-4]. Specifically, difficulties with engagement, recruitment, and retention of racial and ethnic minorities in clinical trials have been described [5-9].

The literature on barriers to minority participation in clinical trials is substantial. These barriers primarily revolve around themes of distrust, lack of interest or awareness in clinical trial participation opportunities, ineffective communication of research findings, and a perceived absence of respect from study team members [10-15]. Conversely, a positive relationship with healthcare providers and an altruistic outlook are frequently cited as motivators for participation. Given the history of unethical human trials in the USA, such as the Tuskegee syphilis study and Puerto Rico contraceptive trials, it is imperative to recognize their lasting impact on the non-Hispanic Black (NHB) and Hispanic communities [15-25].

Merely addressing barriers to minority participation is insufficient. Organizational and provider-level barriers also present significant challenges to inclusive recruitment [26, 27]. Strategies to surmount these include adapting materials to various literacy levels, material translation, recruiting a diverse research staff, and fostering relationships with community organizations that serve underrepresented groups [15, 28].

While there is extensive literature describing the under-representation of minorities in clinical trials, far fewer studies have explored interventions to alleviate this issue. In this study, we sought to increase the proportion of NHB and Hispanic adult patients in the patient population of an ongoing NIH-funded phase II clinical trial (“Randomized-Controlled Trial of Virtual Reality for Chronic Low Back Pain to Improve Patient-Reported Outcomes and Physical Activity,” NCT04409353) [21]. To do this, we developed a multiphase approach: first, we conducted semi-structured focus groups consisting of NHB or Hispanic patients to inquire about possible barriers and facilitators to participation in the parent trial. We then developed culturally tailored patient recruitment materials using these insights. The second phase consisted of improving the diversity of the parent trial’s sample by deploying these new recruitment materials, forming partnerships with relevant communities, and microtargeting patients in the Cedars-Sinai electronic health record.

## Methods

### Phase Ia: Focus Groups

To augment the diversity of the study, our group received supplemental funding from the NIH (UH3AR076573-04S1) to devise and test strategies for enhancing participant diversity. Our supplemental research proposal set recruitment targets designed to mirror the diverse demographics seen in Los Angeles County: going forward, 17% of the study population would be NHB and 40% would be Hispanic.

We used these funds to host focus groups consisting of a NHB and Hispanic individuals identified within the Cedars-Sinai health record. Eligible patients were those aged 13 or older, identifying as non-Hispanic Black (NHB) or Hispanic, and having a documented diagnosis of cLBP lasting more than 3 months [29]. Focus groups were intentionally diverse, spanning differences in age, sex, education, and socioeconomic status. We also intentionally incorporated NHB and Hispanic individuals who had been reached out to in the past for participation in the parent trial, whether they joined the study or not, to incorporate their experiences in the feedback. A total of 25 participants were scheduled to attend, and 13 were present for the sessions (2 NHB, 2 Hispanic; *n* = 13, 2–4 per group).

We conducted 90-min focus groups on Zoom (San Jose, CA: Zoom Video Communications Inc.) to explore participant attitudes toward clinical research. Participants were provided copies of materials used in the parent study, including the informational flyer, recruitment email, user manuals, and consent forms (collectively “study materials,” Appendix 1) 1 week prior, then 24 h prior, to their scheduled focus group for review. Focus group moderators used a guide designed with open-ended, think-aloud exercises and scripted probes to solicit responses from participants. Participants were asked to react to each of the study materials in their own words and without prompting. The moderator followed scripted probes to expand on ideas and specific language introduced by participants. At least one PhD social scientist with qualitative expertise was present to moderate each group.

The audio of each focus group session was recorded with the participants’ consent. Focus group responses were transcribed verbatim (Keystrokes, Santa Monica, CA), and the transcripts were analyzed by two independent coders using an inductive thematic analysis approach. After completing coding, the coders met to extract themes, which were vetted by the full study team. The noted participant suggestions for textual and image changes to recruitment materials were aggregated, and the study team convened to tailor recruitment and other study materials accordingly. The study team adapted materials and implemented the participant suggestions where appropriate and when possible under the authors’ Institutional Review Board (IRB) policies.

To evaluate the revised recruitment materials for clarity and validity, we conducted six follow-up telephone interviews lasting up to 1 h each—three calls were made with NHB and Hispanic participants apiece to confirm the acceptability of the adapted materials. The interviews were not audio recorded but were rigorously transcribed by two investigators, including one with a PhD and qualitative inter-viewing experience.

### Phase Ib: Material Adaptation Strategy

Given the highly overlapping feedback across the NHB and Hispanic cohorts, only a single set of revised study materials was necessary for use across both populations. Table 1 summarizes the specific actions taken to adapt the recruitment and study material in this study and the major themes addressed by each action. Adaptations were IRB-approved (Cedars-Sinai IRB identifier: STUDY00000631) and incorporated into study recruitment on May 15, 2022; at this time, 222 of the eventual 385 study samples had been randomized. The timing of these changes yielded a natural pre-post experiment with comparable population sizes, which we used to test the effectiveness of our changes (collectively the “intervention”).

### Enhanced Communication and Safety Measures

Our study materials were both modified to clarify the 12-week study obligations in response to requests for transparent communication. We also added diverse imagery and QR codes for improved accessibility and highlighted compensation within IRB and ethical limits. Although focus groups suggested emphasizing reimbursement in bold at the beginning of the letter and flyer to pique interest, we instead moved the un-boldened mention of $225 in Amazon e-gift card compensation within the recruitment letter, per IRB guidance. We addressed safety concerns by emphasizing that participation in the parent trial did not involve changes in current pain treatments or the use of experimental drugs.

### Culturally Sensitive Language Revisions

The term “subject” was uniformly replaced with the more acceptable term “participant” throughout all patient-facing study materials. Recruitment letters were rewritten for clarity and brevity, reducing the word count from 360 to 164. The research aims and relevant prior studies were explicitly mentioned.

For example, the phrase “…that seeks to improve chronic low back pain without changing your current treatments or take experimental drugs…” was simplified to:“This study is based on prior findings showing virtual reality's effectiveness in reducing low back pain.”

We also further stressed the voluntary nature of participation to address safety and transparency concerns. Feedback indicated that the original hardware manuals were too thorough and disorganized. Participants described problems with aesthetic clarity and word count: “Because the user guide and everything was so dense… I wish I could watch a video. Like, the idea of being able to fast forward what I already understand, press play. Like, that would be helpful,” FG2-NHB-P1.

### Personalized Recruitment

To address cultural gaps and improve the trustworthiness of our outreach, the study team incorporated a treating physician into our outreach correspondences. The email signatures from 15 Cedars-Sinai Health System physicians with high volumes of patients with cLBP were obtained and inserted into our initial recruitment emails. Following preliminary chart review, these partner physicians were emailed a list of their patients and confirmed which they believed would be eligible for the trial. Study coordinators were trained to mention their physicians had recommended we reach out to them at the start of recruitment phone calls.

The recruitment flyer was adapted to emphasize why a patient would want to join the trial, who the study team members are, and the purpose of the trial. The title on the flyer was changed from “VR for chronic lower back pain reduction study; Randomized control trial of virtual reality for chronic low back pain to potentially reduce pain” to “Do you have low back pain? Virtual Reality for lower back pain study” to address the population of interest rather than describe the intervention.

### Phase Ic: Microtargeting and Community Outreach

The Cedars-Sinai electronic medical record cohort builder was filtered down to create a list of NHB and Hispanic patients meeting the parent study inclusion criteria.

Further, if a patient had visited any of the 15 partner physicians within the past 6 months, they were prioritized for contact, and the name of the most recently visited partner physician was incorporated into outreach. During the post-intervention study period, our team formed partnerships with communities of interest to supplement health record–based recruitment. We partnered with a local branch of pain physicians at the Saban Clinic, a local safety-net provider associated with Cedars-Sinai with a location less than 1 mi, and the U.S. Pain Foundation’s Black, Indigenous, and People of Color (BIPOC) Chronic

### Phase II: Effects on Participant Representation Before vs. After Intervention

The new materials and methods were IRB-approved and integrated into the study recruitment procedures in May 2022 (collectively “study materials,” Appendix 2). The change in pre-intervention to post-intervention randomization success rate, which adjusts for the difference in the volume of patients approached for recruitment, was significantly increased in both the NHB cohort (28.2 to 49.3%, *p* = 0.003) and Hispanic (32.2 to 47.9%, *p* = 0.015) cohorts. Pain Support Group, wherein our revised study flyer was circulated in waiting rooms and support group meetings, respectively. Since these were predominantly not Cedars-Sinai patients, they were required to provide our study team with a signed letter from a physician to confirm an active cLBP diagnosis and were not subjected to the personalized recruitment letter. This requirement was made less burdensome at the Saban clinic by integrating Saban physicians in pre-filled forms and designating a secure area for forms to be dropped off by patients and picked up by study staff.

### Phase II: Quantitative Data Analysis

The number of Hispanic and NHB patients approached for study interest and randomized into the study was aggregated and compared pre- and post-integration of adapted materials and strategic changes using the Chi-squared test or Fisher’s exact test. Additionally, a multivariable logistic regression was fitted for the randomization with an interaction term between race/ethnicity and pre-/post-integration among NH White, Hispanic, and NHB.

## Results

### Phase I: Thematic Analysis

Four focus groups were conducted among NHB (2 groups with *n* = 8 total) and Hispanic (2 groups with *n* = 5 total) participants. The median age of focus group members was 31 years (27–72); gender identities included 7 females and 6 males. Table 2 shows detailed participant characteristics. A total of 6 follow-up interviews were conducted. As no additional actionable suggestions nor critical flaws were identified, thematic saturation was considered reached and materials optimized.

Four key themes emerged from the focus group sessions: (1) mistrust of scientific research; (2) lack of interest; (3) cultural pressures, including unmet language needs of Spanish-speaking patients among Hispanic participants; and (4) communication including lack of transparency. Table 3 shows relevant quotes reflecting these themes.

Overall, the proportion of NHB individuals approached for the parent study out of all individuals approached increased following the intervention, though not significantly (19.0 to 23.5%, *p* = 0.102). However, in the Hispanic cohort, we saw a statistically significant increase (16.6 to 39.9.%, *p* < 0.001). Similarly, the proportion of NHB individuals randomized into the parent study out of all individuals randomized significantly increased following the study intervention, though not significantly, from 16.7 to 20.9% (*p* = 0.295), whereas the increase in the Hispanic cohort was statistically significant (16.7 to 34.4%, *p* < 0.001). Table 4 shows that the logistic regression model showed similar results. The rate of randomization for patients receiving the new modified and personalized recruitment letter was significantly higher in Hispanic patients (6.8 to 39.1%, *p* < 0.001) and higher for NHB patients though not significant (19.6 to 35.8%, *p* = 0.065). Not all participants randomized into the parent study throughout its duration necessarily received a recruitment letter and study flyer by email prior to phone outreach. Individuals who did not receive a recruitment letter—typically patients directly referred to the team through a treating physician or through community outreach—were significantly more likely to enroll in the parent study, with a significantly higher randomization success rate both across NHB participants (41.6% no letter to 29.3% with letter, *p* < 0.029) and Hispanic participants (54.0% no letter vs. 31.7% with letter, *p* < 0.001).

## Discussion

Improving racial and ethnic diversity in clinical trials is not just an ideal but a necessity for minimizing health inequities. Our study sheds light on the barriers to clinical trial participation faced by African American and Hispanic patients while presenting facilitators that, when integrated into recruitment strategies, can significantly improve the probability of an individual choosing to participate in a clinical trial.

This started with a true understanding of the barriers and facilitators of minority clinical trial participation. The focus group sessions revealed four predominant themes—mistrust, interest, culture, and communication—that helped guide the modification of our recruitment strategy and study materials. Mistrust of the healthcare system is an ongoing concern, often cited in existing literature [30]. We therefore made efforts to redesign our recruitment materials, aiming to make them more transparent and focused. We replaced complex language with straightforward explanations, emphasized the safety protocols of our study, and shared findings from previous related research to build trust.

The language and visual elements of the recruitment materials were tailored to reflect the cultural and community norms of our target populations. We did not merely translate our materials but transformed them to resonate with the audience. We incorporated targeted cohort builds from electronic health records and further personalized the recruitment process with letters from treating physicians. These targeted and direct communications helped to establish an immediate rapport and fostered an environment that encouraged participation.

Additionally, previous studies show that sociocultural sensitive mediums improve recruitment when compared to general advertisements [31]. In our study, we changed images in the recruitment flyer and created multimedia materials for the purpose of garnering interest. *Culture* played a significant role in shaping general beliefs about clinical research and highlighted the importance of trustworthy providers as playing a facilitator role in participation. Specifically, it is evident that the need for translation to the Spanish language is of utmost importance to the recruitment of the Hispanic population.

Communication strategies were also enhanced. Based on participant feedback, our updated approach comprised a variety of mediums including digital communication platforms, physical drop boxes, video introductions, and QR codes. This multiplicity of communication modes served to increase engagement and reduce the chances of missed opportunities for recruitment. The relevance of providing clear incentives was also noted and implemented cautiously, ensuring ethical considerations were adhered to.

Taken as a whole, our changes to the recruitment letter, the study flyer, and other materials created statistically significant improvements in the randomization success rate for both cohorts. However, when not adjusting for the total number of participants reached out to, we observed several comparisons where our intervention created significant increases in the Hispanic cohort and insignificant increases in the NHB cohort. We believe that this difference in significance can largely be explained by the timing of the supplementary funding and the amount of opportunity between the pre-intervention performance and the recruitment targets, which were defined before the intervention went into effect in May 2022. By then, the 16.7% Hispanic enrollment rate fell 23.3% below the 40% target we outlined in the research plan; however, the 19.0% NHB recruitment had by then exceeded the 17% recruitment target we had outlined. The statistically insignificant improvement in the second half of the study for the NHB enrollment can be largely attributed to the fact that less recruitment was required to maintain NHB enrollment when compared to the more than double Hispanic randomization that was targeted. Although excluded from our microtargeting efforts, the study team continued to recruit patients who did not fall under the NHB and Hispanic cohorts who primarily heard about the study through community outreach and media. This diminished the overall sample diversity.

Our intervention combined the use of community outreach and health record cohort builds for patient identification. Our results demonstrated that community outreach beyond the immediate health system yielded a superior randomization success rate across both cohorts. However, unless partnerships are formed strictly with underrepresented communities, researchers may find it difficult to balance the pace of recruitment with the diversity of the sample recruited.

Microtargeting, despite being less effective than community outreach, nonetheless, increased the diversity of our study sample. Further, our team completed recruitment without exhausting the number of NHB and Hispanic patients eligible for contact, as there is no shortage of diverse patients with cLBP in the health record of a metropolitan hospital network. This health record-led microtargeting of diverse patients may be limited in other studies based on the number of patients meeting specific study and diversity criteria in the health system. Further, the research budget and duration of the study may incentivize researchers to pursue the traditionally easier recruitment of overrepresented populations to ensure recruitment milestones are met. A hybrid approach which combines community outreach and micro-targeting, such as the one deployed for this trial, can allow researchers to efficiently recruit from the community while leveraging microtargeting when recruitment slows and/or negatively deviates away from diversity targets.

These results highlight the effectiveness of designing recruitment strategies that are not only tailored but are also based on specific, nuanced community needs and preferences. It also emphasizes the importance of proactively revising IRB standards to align better with community needs, when relevant and appropriate.

Although we reached qualitative thematic saturation in this study, this study focused on sampling Black and Hispanic voices in our Southern California community which may not fully reflect perceptions of the patients in other parts of the country. These were also patients who volunteered to participate in a research study, albeit not a clinical trial but were willing to engage with the study staff, which may have introduced self-selection bias. There were participants who were eligible for the primary study and chose not to enroll and who also chose not to participate in the focus groups. These are voices that may have expressed additional barriers, not realized in this study. Because the software used in the study VR device has not yet been translated into Spanish, recommendations to translate materials were noted but could not be implemented by the study team.

In conclusion, enhancing diversity in clinical trials requires intentional, well-informed, and culturally sensitive strategies. An effective approach must involve multiple stakeholders, including but not limited to community leaders, clinicians, and IRBs. It should also be adaptive, rooted in empirical evidence, and, above all, ethical. Therefore, the recruitment strategies for clinical trials should not be considered static but continually evolving to meet community-specific needs and emerging research data.

## Supplementary Material

Supplementary file1

Supplementary file2

**Supplementary Information** The online version contains supplementary material available at https://doi.org/10.1007/s40615-024-02166-y.

## Figures and Tables

**Table 1:** Major themes resulting from analysis of focus group discussions and representative quotations of participants

Theme	Quote(s)
Mistrust of scientific research	“… like about randomized control trial, like for me, like, let's break down those three words. So random. Like that's not it. That's not a word with a positive connotation. Second, the word trial is worse than the word research, in my opinion. It is like a roll the dice. Like, so those three, those two words in close proximity. I'm like, no. And so—” P3G2H “And what about control? How is it controlled? What does control mean? You're going to control me?” P1G4B “… the first thing that I think of is the Tuskegee experiment and feeling unsafe.” P1G2B “I think people of color generically have a mistrust with the medical system, just because of America's history.” P1G2H “So, people hear these things that, you know, a medical group, or doctors are doing research and immediately where I think people go is that, oh, they're making experiments on us like they did down south, or like they did in Puerto Rico, you know, that kind of thing…” P2G1H
Lack of interest in medical research	“…primary motivator probably would be if I thought there was a particular benefit for me that I might experience some kind of relief or alleviation of some symptom or whatever.” P1G4B “I think always, you know, offering a payment. Payment is always, you know, we don't like to talk about it, but money—money does excite people and invites people to do things.” P2G1H “I think what kind of gets in the way is sometimes scheduling. And when I was reading the paperwork, I was like, man, do I have this time to complete [it]?” P2G3H
Cultural pressures to stay away from medical research	“I think there's little interest in the community… It's not in our culture to participate in these things.” P3G1H “I think oftentimes within the Latino community, the lack of psychoeducation… gets in the way.” P2G3H “I think the Hispanic community isn't too gung-ho to get involved in these things, because they feel that they're going to be used as guinea pigs.” P2G1H “…and nobody asking the questions ever looked like me. But they're asking questions to people, to people that look like me, So everyone answering looks like me, but I know that the questions might not have even been formulated with me in mind.” P1G2B
Transparency and appropriate communication	“Not knowing enough information about it, and if there hasn't—I think if it's the first trial being done, I would fear [participating]” P2G3H “I think the thing that first comes to mind is will the views and opinions actually be acted on, right? I think the easy thing is just to collect information. I think the hard thing is actually to take that information and create value out of it.” P2G4B “…how much is it—how much is it going to change my day-to-day life? You know, if it comes to my acupuncture, or my diet, like, do I have to stop eating chocolate?” P3G2B “You know, it’s something about getting that information from someone that the patient respects rather than just hearing it on the corner or whatever. You know it’s, its’s coming from a space of authority, you know? I’m your doctor, I’m taking care of this, but you know, you should also look into this to see if that can help you also. That what I think.” P3G1H “And I was like, Oh my gosh, you know, so the experiment, experience is different when you don’t trust your doctor. But I’ve had doctors that I’m like, I love you, you could tell me to go jump off such and such and I’ll fly, and Imma at least consider it, you know? Definitely, that makes a difference, is trusting.” P1G2B

**Table 2:** Demographic characteristics of participants in focus groups and source of recruitment (*n* = 13)

Patient ID	Age	Sex	Race/ethnicity	Source of recruitment
1	35	M	Hispanic	Deep6 (EHR)
2	61	F	Hispanic	Primary Study
3	67	M	Hispanic	Primary Study
4	31	F	Hispanic	Deep6 (EHR)
5	27	TGF	Hispanic	Deep6 (EHR)
6	53	M	Black	Primary Study
7	31	F	Black	Deep6 (EHR)
8	30	F	Black	Deep6 (EHR)
9	29	F	Black	Deep6 (EHR)
10	72	F	Black	Primary Study
11	29	M	Black	Primary Study
12	58	M	Black	Deep6 (EHR)
13	30	M	Black	Deep6 (EHR)

*M* male, *F* female, *TGF* transgender female, *EHR* electronic medical record

**Table 3:** Summary of study actions to adapt recruitment and study materials and major themes addressed from focus group sessions with Hispanic and Black patients

Adaptation Action	Mistrust	Lack of interest	Cultural pressures	Communication & transparency
Clearly delineated study obligations and any potential side effects	✓			✓
Utilized QR codes for improved accessibility				✓
Highlighted compensation		✓		
Altered the channels of communication to include patients’ providers, community stakeholders, and target-population gatekeepers	✓	✓	✓	✓
Explicitly stated aims of study, study team and pertinent previous research	✓			✓
Used culturally sensitive verbiage & images	✓			✓
Emphasized voluntary nature of research		✓		
Decreased word counts		✓		
Created personalized letters signed by patient’s physicians	✓	✓	✓	
Altered the user manuals with more direct language and visualizations and created a video		✓		✓

**Table 4:** Table of logistic regression results for the randomization rates by race for pre- and post-adaptation of study materials

	Odds ratio	95% CI	*p*-value
NH White Post (v. Pre)	5.71	3.22, 10.12	< .001
Hispanic Post (v. Pre)	1.84	1.14, 3.30	0.015
NH Black Post (v. Pre)	2.47	1.35, 4.52	0.003

## Data Availability

Data including focus group transcripts and recruitment statistics are stored on a secure “Box” file and can be provided upon request.
