# Supplementary material for: Bridging the Gap: Culturally Responsive Strategies for NIH Trial Recruitment
Source: J Racial Ethn Health Disparities. Author manuscript; Available in PMC 2025 Dec 5. (PMC12644225; doi:10.1007/s40615-024-02166-y)
Supplement: Supplementary file1 [file NIHMS2122634-supplement-Supplementary_file1.pdf]

Subject: Information about Cedars-Sinai Lower Back Pain Study

Cedars Sinai

[Date]

Dear [Patient Name],

In addition to the medical care provided at Cedars-Sinai, we conduct research to learn how to better prevent, diagnose and treat illness, with the ultimate goal of improving health.

As the Director of Health Service Research at Cedars-Sinai, I am conducting a research study, **Randomized-Controlled Trial of Virtual Reality for Chronic Low Back Pain to Improve Patient-Reported Outcomes and Physical Activity**, with the purpose of seeing if and how virtual reality can improve chronic pain and physical function. I am contacting you to invite you to consider participating in this study.

Participation in research is completely voluntary. Your decision to participate or not will not affect your medical care at Cedars-Sinai. If you do not wish to be contacted further about this study or would like to decline participation, please call or email Joshua Fouladian, at 310-423-6740 or at VRstudymail@cshs.org. We want to understand your reasons for declining participation, so please also include a brief explanation of why you do not want to participate. Otherwise, a member of the research team will contact you in about one week to see if you are interested in learning more about this study.

If you are found to be eligible and decide to participate, you will be asked to complete multiple online surveys over the course of the 90-day study period. After completing a

set of surveys, you will receive a virtual reality headset, a Fitbit device for the duration of the study.

Individuals who choose to enroll may be eligible for up to a $225 amazon gift card at the completion of the study after returning the equipment based on the number of surveys completed. To ensure the safety of our participants, this study is being conducted remotely.

The research will be conducted remotely using email or text-based surveys. For more information, please contact us at VRstudymail@cshs.org to learn more about the study, our team, and research at Cedars-Sinai.

Sincerely,

Principal Investigator Signature

Title I

Title II

Title III

Cedars Sinai

VR FOR CHRONIC LOWER BACK PAIN REDUCTION STUDY

Randomized-controlled trial of virtual reality for chronic low back pain to potentially reduce pain

A Cedars-Sinai Research Project

WHO ARE WE?

We are a research group at Cedars-Sinai interested in studying how pain affects quality of life. We are working closely with people who have chronic lower back pain to determine if digital technologies can help improve daily function and reduce pain.

WHAT IS THE PURPOSE OF THIS RESEARCH PROJECT?

Cedars-Sinai is conducting an at-home research study to test whether a virtual reality (VR) headset can help manage or reduce chronic lower back pain.

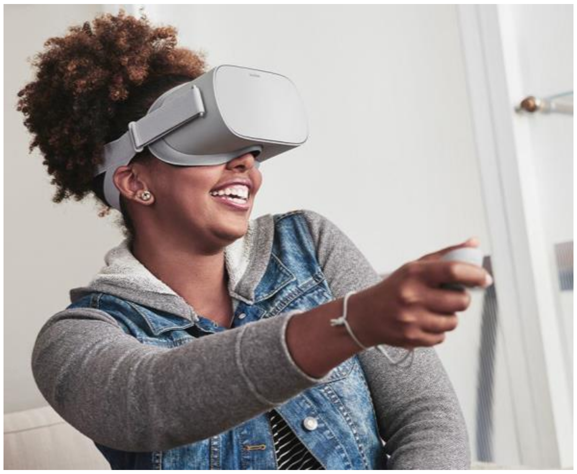

WHO IS ELIGIBLE?

For this study, we are looking for patients who:

- are age 13+
- have at least 3 months of persistent low back pain
- have felt almost daily pain over the past 6 months
- own a computer or smartphone with access to email

HOW CAN I PARTICIPATE?

- If you meet the criteria above, please contact us using the below contact information
- A study team member will find a time to assess your eligibility over the phone
- After you complete a week of surveys, a VR headset and Fitbit Charge 4 will be sent to your home

WHAT CAN I EXPECT IF I PARTICIPATE?

- **The study does not require any in-person medical visits**
- You will be eligible for up to $225 in Amazon gift card codes
- You will be asked to use the VR headset at least once daily and to wear a Fitbit activity tracker
- You will answer **survey questions** about your pain on a weekly basis over 12 weeks
- Your participation may provide benefits such as **pain relief** and improved **physical ability**
- All devices will be returned to the study team at the end of the study

For more information about the study, please contact the VR Study Team or visit our website by scanning the QR code with your smartphone camera.

Phone: 310-423-6740
Email: VRstudymail@cshs.org
