# Supplementary material for: Bridging the Gap: Culturally Responsive Strategies for NIH Trial Recruitment
Source: J Racial Ethn Health Disparities. Author manuscript; Available in PMC 2025 Dec 5. (PMC12644225; doi:10.1007/s40615-024-02166-y)
Supplement: Supplementary file2 [file NIHMS2122634-supplement-Supplementary_file2.pdf]

At-Home Virtual Reality for Lower Back Pain
An Invitation to Participate in a Cedars-Sinai Study

Subject Line: At-Home Virtual Reality for Lower Back Pain: A Cedars-Sinai Study Invitation

Dear [*Patient name*],

I am writing to follow up on our previous visit and share some information about an exciting, new, non-invasive study being conducted at Cedars-Sinai, with sponsorship by The National Institutes of Health (NIH), that seeks to improve chronic low back pain **without the need to change your current pain treatments or take experimental drugs**. My colleague, Dr. Brennan Spiegel, and his team are investigating whether using a virtual reality (VR) headset with specialized programming can improve chronic low back pain. This study is based on previous findings that showed virtual reality was effective in reducing low back pain and pain from other conditions.

Participants are eligible for up to $225 in Amazon e-cards. The study spans 12 weeks, and if you decide to participate and are eligible, you will be asked to use the VR headset for a short time each day and complete online surveys every week. This study is entirely remote, without any need to visit the hospital, clinic, or any doctors in person; all study equipment is provided by mail at no cost. The study is titled, *Randomized-Controlled Trial of Virtual Reality for Chronic Low Back Pain to Improve Patient-Reported Outcomes and Physical Activity*. I feel you are a good candidate for this study and may benefit from participating if you qualify.

That said, your choice to participate is a personal decision, and it will have no effect on your care at Cedars-Sinai. In fact, participation is completely voluntary, and you can choose to withdraw from the study at any time and for any reason. For more detailed information about this study, you can follow this link. A member Dr. Spiegel's team will contact you after 7 days to see if you are interested in participating and to answer any questions you may have.

You may contact his team by email at VRstudymail@cshs.org or by phone at 310-423-6740.

In addition to the medical care provided at Cedars-Sinai, we conduct research to learn how to better prevent, diagnose and treat illness, with the ultimate goal of improving the health and quality of life for our patients and community.

Sincerely,

Physician Signature
Physician Title I
Physician Title II
Physician Title III

PROPER PHYSICIAN ADDRESS

Cedars Sinai

Do you have low back pain?

Virtual Reality for Lower Back Pain Reduction Study

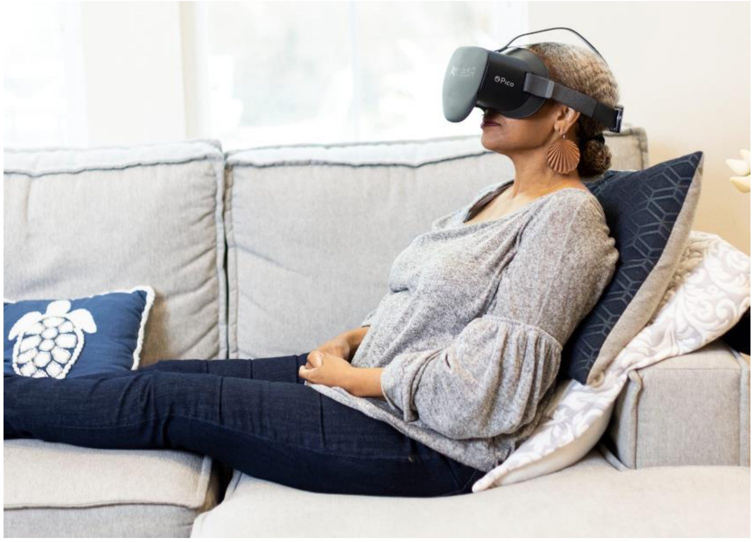

Purpose

Cedars-Sinai is conducting an at-home study to test whether using a virtual reality (VR) headset with specialized programming for a short time every day can help manage or reduce chronic lower back pain. VR allows the wearer to experience different environments in the comfort of your home.

Who is eligible?

For this study, we are looking for patients from diverse backgrounds who:

- Are age 13+
- Have felt low back pain over the past 6 months
- Own a computer or smartphone with access to email

What can I expect if I participate?

- The study does not require any in-person medical visits.
- Up to $225 in Amazon e-cards.
- Use a VR headset at least once daily.
- You will answer surveys about your pain over 12 weeks.
- Your participation may provide benefits such as pain relief and improved physical function
- No changes to your current pain treatments or use of experimental drugs

For more information, contact the VR study team or visit our website by opening your smartphone's camera app and focusing on the QR code.

Phone: 310-423-6740

Email: VRstudymail@cshs.org
