# Supplementary material for: Bridging the Gap: Culturally Responsive Strategies for NIH Trial Recruitment
Source: J Racial Ethn Health Disparities. 2024 Oct 29;12(6):3655–63. doi: 10.1007/s40615-024-02166-y (PMC12644225; doi:10.1007/s40615-024-02166-y)
Supplement: Supplementary file 1 [file 40615_2024_2166_MOESM1_ESM.docx]

Technical Appendix 1 (Pre-intervention)

1.1 Recruitment Email

1.2 Informational Flyer

1.3 Device Delivery Email

1.4 VR Headset User Guide

1.5 Frequently asked Questions
