# Supplementary material for: Bridging the Gap: Culturally Responsive Strategies for NIH Trial Recruitment
Source: J Racial Ethn Health Disparities. 2024 Oct 29;12(6):3655–63. doi: 10.1007/s40615-024-02166-y (PMC12644225; doi:10.1007/s40615-024-02166-y)
Supplement: Supplementary file 2 [file 40615_2024_2166_MOESM2_ESM.docx]

Technical Appendix 2 (Post-intervention)

2.1 Recruitment Email

2.2 Informational Flyer

2.3 Device Delivery Email

2.4 VR Headset User Guide
